# Human subthalamic nucleus neurons differentially encode speech and limb movement

**DOI:** 10.3389/fnhum.2023.962909

**Published:** 2023-02-17

**Authors:** Karim Johari, Ryan M. Kelley, Kris Tjaden, Charity G. Patterson, Andrea H. Rohl, Joel I. Berger, Daniel M. Corcos, Jeremy D. W. Greenlee

**Affiliations:** ^1^Human Neurophysiology and Neuromodulation Lab, Department of Communication Science and Disorders, Louisiana State University, Baton Rouge, LA, United States; ^2^Department of Neurosurgery, The University of Iowa, Iowa City, IA, United States; ^3^Medical Scientist Training Program, The University of Iowa, Iowa City, IA, United States; ^4^Program in Neuroscience, The University of Iowa, Iowa City, IA, United States; ^5^Department of Communicative Disorders and Sciences, University at Buffalo, Buffalo, NY, United States; ^6^Department of Physical Therapy, University of Pittsburgh, Pittsburgh, PA, United States; ^7^Department of Physical Therapy & Human Movement Sciences, Northwestern University, Chicago, IL, United States; ^8^Iowa Neuroscience Institute, Iowa City, IA, United States

**Keywords:** Parkinson’s disease, vocalization, diadochokinesia, single unit, multi-unit

## Abstract

Deep brain stimulation (DBS) of the subthalamic nucleus (STN), which consistently improves limb motor functions, shows mixed effects on speech functions in Parkinson’s disease (PD). One possible explanation for this discrepancy is that STN neurons may differentially encode speech and limb movement. However, this hypothesis has not yet been tested. We examined how STN is modulated by limb movement and speech by recording 69 single- and multi-unit neuronal clusters in 12 intraoperative PD patients. Our findings indicated: (1) diverse patterns of modulation in neuronal firing rates in STN for speech and limb movement; (2) a higher number of STN neurons were modulated by speech vs. limb movement; (3) an overall increase in neuronal firing rates for speech vs. limb movement; and (4) participants with longer disease duration had higher firing rates. These data provide new insights into the role of STN neurons in speech and limb movement.

## Introduction

Speech impairment impacts ∼90% of people with Parkinson’s disease (PD) at some point in their disease course (Duffy, 2013; Sapir, 2014). Commonly used PD treatments that improve limb motor function are ineffective for improving speech function. For example, dopaminergic medications are recognized to have highly variable effects on acoustic and perceptual speech measures (e.g., Plowman-Prine et al., 2009; Pinho et al., 2018). Similarly, deep brain stimulation (DBS) of the subthalamic nucleus (STN) consistently improves limb motor symptoms (Deuschl et al., 2006; Weaver et al., 2009) yet has mixed effects on speech outcome, with some studies showing exacerbated speech impairments following STN-DBS (Skodda, 2012; Aldridge et al., 2016). Previous reports on STN-DBS-induced speech impairment have investigated the effect of stimulation parameters (Tornqvist et al., 2005; Dayal et al., 2017; Knowles et al., 2018), stimulation-on vs. stimulation-off (Homer et al., 2012; Sauvageau et al., 2014), pre-stimulation vs. post-stimulation (Tripoliti et al., 2011, 2014), laterality (Wang et al., 2006; Schulz et al., 2012), and electrode position (Tripoliti et al., 2008; Ehlen et al., 2014; Jorge et al., 2020).

Taken together, the inability of medical and surgical PD treatments to reliably improve speech function suggests differential underlying neural mechanisms between limb motor and speech motor functions. One possible mechanism is that STN neurons may differentially encode speech vs. limb movement. To date, this hypothesis remains untested.

Intraoperative recording from human STN during awake DBS surgery provides a unique opportunity to investigate the neural activity of single and multi-neuronal units in response to speech and limb movement. Previous studies have reported modulation of STN neurons, i.e., changes in firing rates, in response to limb movement (Potter-Nerger et al., 2017; Tankus et al., 2017; London et al., 2021). Potter-Nerger et al. (2017) found that 75 of 114 isolated single units within STN showed movement related changes during a reach-to-grasp task. Tankus et al. (2017) isolated 89 single neurons in 10 PD patients of which 38 units were modulated by upper and lower limb movement. London et al. (2021) recorded 39 units from STN in 8 PD patients and demonstrated that a distinct population of neurons (i.e., multi-units) were modulated by movement direction.

Similarly, studies report the modulation of STN neurons in response to speech tasks (Watson and Montgomery, 2006; Lipski et al., 2018; Tankus and Fried, 2019; Tankus et al., 2021). Tankus et al. (2021) recorded 180 neurons in 18 PD patients; 124 of the neurons were modulated by different vowel productions. Lipski et al. (2018) identified 79 neurons (25 single, 54 multi-units) within bilateral STN, nearly of half of which were modulated by single word production. They found that STN neurons had heterogenous patterns of neural firing (increased, decreased, and mixed) in response to the single word production task. STN neurons have also been reported to be modulated by a more complex speech task (sentence production) in a previous study where 21 (out of 35) STN neurons in 7 PD patients were modulated by that task (Watson and Montgomery, 2006). Overall, these reports show that STN neuronal activity (both single and multi units) is modified by production of simple and more complex speech.

Although the extant literature provides some insight into the role of STN in speech and limb movement, we are not aware of any study that directly compare how STN neurons encode speech vs. limb movement. Identification of differential modulation of STN neurons during speech and limb movement may eventually lead to a mechanistic understanding of the contrasting effects of dopaminergic medications and STN-DBS on speech- and limb-motor output and outcomes. Here, we recorded neural activity within right and left STN from 12 non-demented PD patients during awake DBS surgery while they were performing interleaved speech and limb movement tasks. The primary goal of the present study was to determine if there are differences in patterns of neuronal firing within STN for speech and limb movement during microelectrode recording (MER)-guided DBS implantation surgery. The secondary goal was to determine if STN firing rates during these tasks are affected by factors related to PD. For example, a previous study indicated that STN neuronal firing rates increased with PD progression (Remple et al., 2011). However, it is unclear to what extent the neural firing rates in response to speech and limb movement may correlate with patients’ clinical characteristics such as disease duration and PD factors. We explored the possible association between task-related modulation of STN neurons and measures of dopaminergic sensitivity and disease duration.

## Materials and methods

### Participants

Data from 12 participants with PD (4 females; mean age = 64.6 ± 1.5 years) undergoing bilateral STN-DBS implantation surgery were included (see Table 1). All participants completed an extensive pre-surgical assessment including detailed neurological examination, structural MRI, levodopa challenge and formal neuropsychological evaluations. They participated under procedures approved by the University of Iowa’s Institutional Review Board, and all provided written informed consent prior to study participation.

**TABLE 1:** Demographics and PD characteristics of 12 participants reported.

Subject ID	Age (years)/Sex	Handedness	PD Duration (years)	Neurons (R STN/L STN)	UPDRS III: Total (OFF/ON)*	UPDRS III: Speech (OFF/ON)*	UPDRS III: Finger bradykinesia (OFF/ON)*
253	67/F	Right	17	5/0	50/10	1.5/0.5	Right: 1.5/0
							Left: 2.5/1.5
261	59/F	Right	8	4/6	35/13	1/0	Right: 1/0
							Left: 2/1
265	66/M	Right	6	0/5	21/11	0/0	Right:1/0
							Left: 0.5/0.5
269	62/M	Right	9	4/3	36/19	1/1	Right: 2/1
							Left: 1/0.5
270	52/M	Right	6	2/2	48/26	3/1	Right: 3/2.5
							Left: 2/2
273	71/M	Left	9	3/4	38/19	1/1	Right: 1/1
							Left: 2/1
274	62/M	Right	7	4/3	37.5/8	0/0	Right: 1/0.5
							Left: 3/1
276	67/M	Right	4	0/5	67.5/47	1/0	Right: 2.5/1
							Left: 4/3
277	65/F	Right	11	3/5	32/9	1/0	Right: 1.5/0
							Left: 2/0.5
278	70/F	Right	2	5/0	50/29	1/1	Right: 1/1
							Left: 1/1
281	65/M	Right	11	4/0	63.5/16	2/1	Right: 2.5/0
							Left: 3.5/0
283	69/M	Right	12	2/0	32/8	1/0	Right: 1/0
							Left: n/a

Denotes Unified Parkinson’s Disease Rating Scale (UPDRS) scores measured preoperatively OFF and ON their usual PD medications.

### DBS surgery

STN-DBS implantation was performed such that bilateral DBS electrodes were implanted sequentially during a single stereotactic procedure. Indirect framed stereotactic targeting of the STN was refined by multielectrode recordings from 0.4 to 0.8 MΩ tungsten microelectrodes (Alpha Omega Co., Inc., Alpharetta, GA, USA). Three to five MER tracks were used for each hemisphere, typically consisting of three simultaneous (anterior, middle, and posterior) trajectories each separated by 2 mm center-to-center. These simultaneous trajectories allowed detailed (i.e., sub-millimetric) sampling of the anterior/posterior and dorsal/ventral axes of STN. STN margins were defined by the functional and electrical properties from STN clinical MER, including presence of large and medium amplitude single units demonstrating burst, pause, or tremor related activity, consistent with standard reported techniques with current commercial software (e.g., Alpha Omega Co., Inc.; for similar studies see: Benazzouz et al., 2002; Cuny et al., 2002; Gross et al., 2006; Thompson et al., 2018). Behavioral and neurophysiological data were acquired simultaneously using a Tucker-Davis Technologies (TDT) multi-channel recording system (System 3, Tucker-Davis Technologies, Alachua, FL, USA). MERs were sampled at 24 kHz, amplified and passed from the Alpha Omega system into the TDT system so that spike, voice, and limb movement data had a common timescale.

Because MERs are clinically informative, participants were not exposed to extra electrode penetrations to participate in this research. Surgery was typically prolonged by < 15 min per hemisphere to conduct speech and limb testing. Participants did receive analgesic and sedative medications in the operating room; these were short-acting agents and were stopped > 1 h prior to MER, allowing participants to be maximally awake for necessary clinical testing and research. All participants were off PD medications for ∼12 h prior to participation.

### Localization of MER

Electrode locations within the physiologically defined boundaries of STN were included in the study. Since multiple simultaneous microelectrodes were utilized, recording locations for each participant were determined relative to the dorsal border of STN. All recording locations for this study were localized to the dorsal half of STN as this is the accepted motor subregion of STN and target for DBS leads. Intraoperative time constraints precluded receptive field mapping of STN neurons (e.g., face versus limb vs. potential speech responsive intra-STN differences). Each participant had at least two microelectrodes in dorsal STN during experiments. Per clinical protocol, we did not routinely probe the medial/lateral STN axis, nor did our clinical protocol include routine post-operative brain imaging. Therefore, we are unable to comment on the relative medial/lateral distribution of recording locations within STN.

### Behavioral testing for speech and limb function during DBS surgery

**FIGURE 1:**
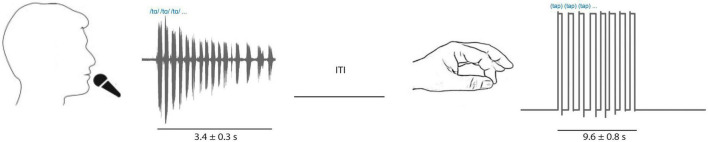
Tasks included interleaved speech (sustained vowel phonation [/ɑ/] and diadochokinetic [/tɑtɑtɑ/]) repetitions captured by a microphone (**left**; example/tɑtɑtɑ/audio signal shown) and limb movement trials (**right**; contralateral finger tapping, example goniometer tracing shown). Inter trial interval was variable.

Once microelectrodes were positioned within the physiologically defined STN, participants performed interleaved trials of speech and limb motor tasks (Figure 1). Participants received a verbal cue from the experimenter instructing them to either speak or tap their finger. Speech consisted of repetitions (3.4 ± 0.3 s mean duration) of one of two tokens often used in PD clinical speech evaluation: diadochokinesis (/tɑtɑtɑ/) and sustained phonation (/ɑ/). Speech tokens were interleaved throughout ∼10 min recording sessions to produce roughly equal amounts of/tɑtɑtɑ/and/ɑ/trials. Participants were told to speak at a rate and loudness that they would use in everyday conversation. Speech signals were captured *via* a condenser microphone (Beta 87A, Shure, Inc, Niles, IL, USA) positioned close to the mouth, amplified (Audio Buddy, M-Audio LLC, Cumberland, RI) and recorded with MERs using a common time scale. Speech onsets and offsets were detected visually in Matlab from the recorded speech signal.

The limb motor task consisted of blocks of repetitive finger tapping (9.6 ± 0.8 s mean duration) and was captured with a goniometer (SG65, Biometrics Ltd, Newport, UK) fixed to the thumb and first finger. Participants performed finger tapping using the hand contralateral to the STN recorded. Onset and offset of finger tapping were derived from the goniometer signal reflecting when recorded amplitudes deviated from background levels visually in Matlab. The speech and finger tasks were interleaved and chosen to provide short duration, high-yield tasks to assess speech and limb motor function yet provide enough repetitions to allow averaging and correlation with neuronal firing rates.

### Analysis of intraoperative MER data

Raw voltage time series data were sampled from STN MERs at 24 kHz and high pass filtered at 2 kHz to lessen contamination by speech or movement artifacts. The choice of this high pass filter was due to occasional presence of microphonic artifacts below 2 kHz. In a recent study, we showed that microelectrodes that are used to collect spike/LFP data are susceptible to microphonic artifacts (Berger et al., 2022). Although a high pass of 2 kHz can distort the normal spike waveforms and potentially impact signal-to-noise ratio (SNR) and by discarding spikes with dominant frequencies below 2 kHz, it ensures that the data were not substantially contaminated by microphonic artifacts and in our previous study the majority of genuine physiological spikes remained identifiable. Prior to filtering, we used the demodulated band transform (DBT) method to detect and remove transient noise and artifacts from the filtered signal (Kovach and Gander, 2016). In addition to filtering, two additional techniques were utilized to ensure adequate MER quality and mitigate potential contamination by electromechanical artifacts. First, visual inspection was performed and trials containing any remaining microphonic artifacts arising from speech trials were excluded from analysis. Second, trials were evaluated for waveform instability, or non-stationarity. Non-stationarity can result from decreased spike amplitude at high firing rates (Fee et al., 1996; Stratton et al., 2012) or sub-millimetric displacement of the MER during heartbeat and respiration (Joshua et al., 2007). Units affected by non-stationarity appear as multiple smeared clusters in principle component space yet display characteristic inter-spike interval distributions consistent with single units (Fee et al., 1996; Snider and Bonds, 1998). We hypothesized that non-stationarity trials might occur more frequently during tasks due to head movement and vibration, and therefore tested whether louder speech could increase waveform non-stationarity due to speech-induced vibrations causing the STN microelectrode to be momentarily displaced relative to its neuronal target.

To assess the degree of speech-locked non-stationarity in our presumptive single units with respect to voice loudness, root mean square values (RMS) were calculated. RMS is defined as $\sqrt{\left[\frac{\sum_{1}^{n}x_{i}^{2}}{n}\right]}$in which *x_i_* represents a voltage sample. For RMS_signal_, *x_i_* was comprised of the samples recorded in a time window (−0.5 ms to +1.2 ms] surrounding the threshold crossing of *n* spikes of a presumptive single unit. For RMS_sound_, *x_i_* was comprised of the speech audio samples recorded in the same time window (−0.5 ms to +1.2 ms from spike detection) used in RMS_signal_. RMS_sound_ was calculated to determine the correlation of the amplitude of audio waveforms captured by the condenser microphone (i.e., voice loudness) with the peak-to-trough amplitude of spike waveforms. RMS_signal_ was regressed over RMS_*sound*_ to determine if increased voice loudness coincided with a nonstationary spike waveform. Following the method of Stratton et al. (2012), SNRs were calculated as a ratio of RMS values (RMS_signal_/RMS_noise_; RMS_noise_ was calculated from the entire recording after extracted spikes removed) for each presumptive single unit and found to be satisfactory given the neuronal density of STN: (mean 2.7 +/−0.8, range 1.8–5.1; Zwirner et al., 2017).

**FIGURE 2:**
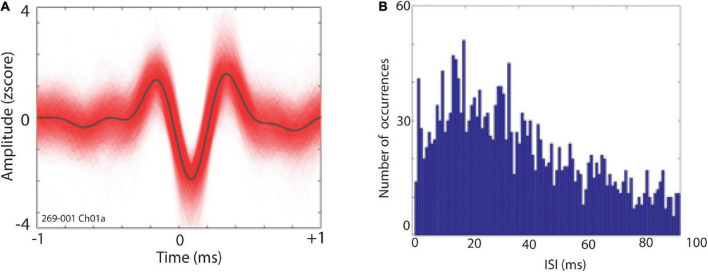
**(A)** Exemplar waveforms of a putative STN single neuron. **(B)** Distribution of interspike intervals (ISI) for single unit depicted in panel **(A)**.

After rejection of contaminated MERs based on post-filtering visual inspection and assessment of non-stationarity, offline spike detection was performed by thresholding at ± ∼3 std. Spikes were sorted using principal component analyses (PCA) in Plexon Offline Sorter (Plexon, Inc., Dallas, TX). Single units were identified as having (i) consistent waveform shape; (ii) separable clusters in principal component space; and (iii) a consistent refractory period of at least 1 ms in interspike interval (ISI) histograms. Figure 2 shows an example of putative single neuron waveforms and ISI distributions. We further quantified the quality of our single units clusters using isolation distances (Joshua et al., 2007) and refractory period violation (Hill et al., 2011). Isolation distance (IsoD), an index of clustering quality for single units, was calculated for single units clusters in Plexon (Joshua et al., 2007). The mean IsoD index wherein more than one unit was recorded on a single channel (*n* = 8) was 24.97 in PCA space. Moreover, refractory period violation was calculated for all 28 single neuron clusters (for more details, see: Hill et al., 2011). We found that these neurons had a mean of 5.06% of spikes occurring within 3 ms of each other. Additionally, a more stringent cutoff (ISIs < 1 ms) for refractory period violation showed that 0.007 percent spikes occurred within 1 ms each other.

Spike times from individual neurons were examined relative to the onset and offset of speech and limb movement using the co-recorded speech and goniometer recordings, respectively. Spike-time activity was aligned according to trial onset or offset, followed by binning to create peri-event time histograms (PETH). PETH data were constructed for each neuron and task.

As others have reported, STN neurons have demonstrated task-related modulations at both single and multi-unit levels (Tankus et al., 2017; Lipski et al., 2018; London et al., 2021). Hence, multi-unit analyses were performed here to investigate the modulations of neuronal clusters at a larger population level for speech and limb movement. All parameters for multi-unit sorting were similar to the single unit analysis. MountainSort toolbox was used to perform multi-unit analysis (Chung et al., 2017). Briefly, data were first bandpass filtered between 2,000 and 6,000 Hz and whitened for spike waveform detection, and then run through the MountainSort algorithm. This offers a fully automated, fast approach to spike detection, involving PCA following amplitude thresholding and a proprietary algorithm for clustering [(ISO-SPLIT) Magland and Barnett, 2015]. Sorted spikes were imported into Matlab for visualization and manual curation. Clusters were labeled as multi-unit if more than one percent of spikes had an interspike interval < 1 ms, consistent with our single unit analyses and others (e.g., Roy and Wang, 2012).

### Statistical analysis

Absolute firing rate in spikes per second (Hz) and response modulation index (RMI) were used to quantify neuronal patterns in STN and determine speech and limb movement-related changes. Firing rate was calculated on a single trial basis and was calculated by dividing total spikes during a given trial by trial length in seconds (*Hz* = spikes/s). RMI, as a measure of percent change in firing between two conditions, was used to assess firing rate modulation occurring around behavioral events (Eliades and Wang, 2003, 2008; Eliades and Tsunada, 2018). Note absolute firing rate is a complementary and more general measure of firing rate changes following the onset of the speech and limb task. RMI was used as a normalized index which can measure more transient modulations around the onset or offset of the events. RMI was calculated using the formula RMI = (R_interval_ –R_baseline_)/(R_interval_+ R_baseline_) where R_baseline_ is spikes per second pre-event (−0.5 to 0 s) and R_interval_ is spikes per second post-event-i.e., after task started (onset) or stopped (offset) (0 to +0.5 s). Baseline for speech and limb offset was 0.5 before the offset of the task. RMI was used to analyze STN neuronal modulation occurring around task onset and task offset, i.e., the time at which subjects started or stopped a given trial. Trials were excluded from RMI analysis if R_baseline_ contained any carryover task performance (e.g., speech occurring during the R_baseline_ period of a limb movement trial, or vice versa). Additionally, we visually inspected trials for both tasks and excluded any trial that had overlap with the baseline of the next trial. RMI > 0 indicates *increased* firing rate during a behavioral event and RMI < 0 indicates *decreased* firing rate. RMI values were calculated around two behavioral events (task onset: RMI_onset_; task offset: RMI_offset_) for each task (speech and limb movement trials).

RMIs and absolute firing rates were separately submitted to a linear mixed effects model with task (speech vs. limb movement), epoch (trial onset vs. trial offset for RMIs), and interaction of task × epoch as fixed effects, and subjects and units as random effects. We used Satterthwaite approximation as the estimation method for degrees of freedom and variances. Note, we did not see significant differences between single and multi-unit firing rates [*t* (125.95) = 0.69, *p* = 0.49] and RMIs [*t* (64.33) = −0.39, *p* = 0.70] during our tasks; therefore we combined our single and multi-units and performed statistical analysis on all 69 STN units except for correlations with clinical PD measures where single and multi-units were analyzed separately as detailed below.

To delineate the characteristics of neuronal units in the current study, we classified identified units into 4 categories based on their RMIs: units that were modulated by (1) speech onset or offset only (2) limb onset or offset only, (3) both speech and limb movement onset or offset, and (4) neither speech nor limb movement. To label units as modulated or not modulated by tasks, first RMIs were calculated at the single trial level, then the 95% confidence interval of the mean RMI was obtained for each of 69 units. We tested mean RMI of each unit against the null value of 0. When the null value 0 was not within the 95% confidence interval limits of RMI distribution, units were labeled as “modulated.” Next, units classified as modulated were submitted to a logistic mixed effects model with task (speech vs. limb movement), epoch (onset vs. offset), and their interaction as fixed effects. Additionally, we adopted subjects and units as random effects since measures were repeated across subjects and units.

Linear mixed effects modeling was performed to determine if four selected clinical variables interacted with speech and limb movement-related firing rate or RMI differences: Unified Parkinson’s Disease Rating Scale (UPDRS) and disease duration. These pre-operative clinical variables were 1. Δ UPDRS III _total_; 2. Δ UPDRS III _speech_; 3. Δ UPDRS III _fingerbradykinesia_; 4. PD disease duration (i.e., time since PD diagnosis). Δ UPDRS III refers to the difference in OFF vs. ON levodopa scores in preoperative UPRDS Part III motor testing; higher UPDRS scores indicate greater impairment. Participants with greater Δ UPDRS III values showed greater overall improvement of PD-related motor impairments when administered levodopa medications during preoperative testing; these delta metrics are measures of levodopa sensitivity. We also performed Pearson correlation analyses between clinical measures of PD and RMIs and absolute firing rates separately for single and multi-units. The rationale for this correlation was based a previous study that showed longer PD duration was associated with increases in the firing rates of single neurons in STN (Remple et al., 2011). We are not aware of any study reporting correlation between multi-units and PD duration. All hypothesis testing was two-sided at α = 0.05 and statistical analyses were conducted in R and Matlab.

## Results

A total of 69 single- and multi-unit clusters (36 R STN; 33 L STN) were identified from 12 participants (see Table 1).

**FIGURE 3:**
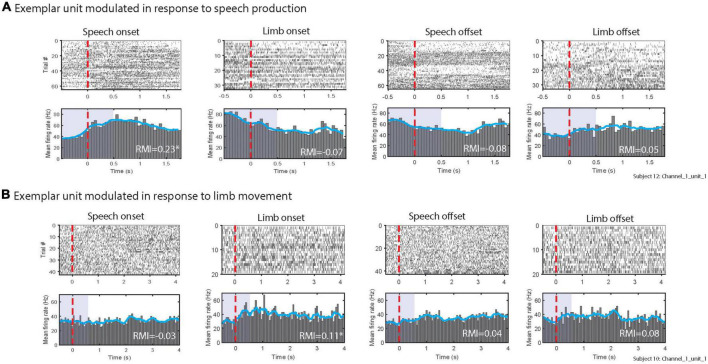
Exemplary multi-unit clusters firing patterns in response to **(A)** speech, **(B)** limb movement. The top rows of **(A,B)** show raster plots of spikes during individual trials. Bottom rows of panels **(A,B)** illustrate peri-stimulus time histograms. Blue lines in histograms display the smoothed mean of firing rates. The red vertical lines show the speech and limb movement onsets (left panels) and offsets (right panels). RMI values for each alignment are listed; * indicates units were significantly modulated as 95% confidence interval did not include 0. Purple shaded areas in peri-stimulus time histograms indicates the time windows used to calculate RMIs.

STN neuronal firing rates were examined in both task onset- and task offset-aligned epochs. Figure 3A shows task onset-aligned (left panels) and offset-aligned (right panels) datasets from an exemplar multi-unit cluster that demonstrated a prominent increase in firing in STN during speech while it showed decreased mean firing rate following limb movement onset; this unit did not show modulation around offset of speech and limb movement. Figure 3B shows a different exemplary multi-unit cluster which did not demonstrate strong modulations for the speech task (neither onset nor offset) but did show increase in the firing rate following limb onset; no large modulation was noted aligned to limb offset.

**FIGURE 4:**
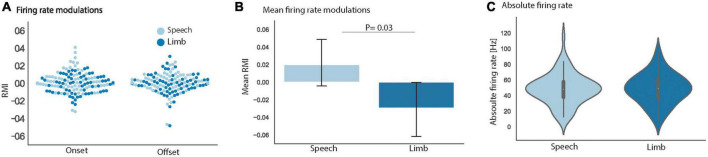
**(A)** Mean RMI for all 69 units separately for the onset and offset alignments of speech (light blue) and limb movement (dark blue) did not differ. **(B)** Group mean RMIs for speech and limb movement tasks significantly differed [*t* (201.40) = –2.18, *p* = 0.03] when onset and offset alignments were combined. **(C)** Group mean absolute firing rates for speech and limb movement did not significantly differ. The vertical lines display ± 1 standard error of the means. Data shown is from all 69 STN neuronal clusters in all panels.

To explore these varied patterns of STN modulation, group-level analyses were done to examine speech- and limb movement-related firing patterns across all 69 identified clusters. RMI and absolute firing rate (Hz) metrics were used to quantify neuronal firing. Whereas absolute firing rate was calculated over the full duration of a trial, RMI identified firing rate modulations in discrete time windows around a behavioral event, in this case task onset and task offset. Figure 4A shows the RMIs for onset and offset alignments of speech and limb movement. Our linear mixed effects model showed no significant interaction between task (i.e., speech vs. limb) and trial type (i.e., onset vs. offset alignment) [*t* (202.01) = 1.56, *p* = 0.14] to indicate an effect of onset or offset on the difference between speech versus limb RMI. There was found to be a significant effect of task with an increase in RMIs following speech compared to a decrease during limb movement [*t* (201.40) = −2.18, *p* = 0.03; Figure 4B] when both task onset and offset alignments were included.

In addition, the group of 69 STN clusters had similar absolute firing rates during speech trials (mean 41.9 Hz) versus limb movement trials [mean 42.1 Hz; *t* (67.44) = 0.41, *p* = 0.68; Figure 4C]. Normalized Z-score comparisons of trial firing rates versus baseline also did not differ between tasks. Notably we did not observe differences in STN firing rates between our speech tokens (/tɑtɑtɑ/vs./ɑ/); therefore, all speech vs. limb comparative analyses were done using pooled speech trials.

We additionally classified neuronal clusters as “modulated” by tasks based on those clusters that had RMI statistically different than zero (see section “Materials and methods”). Using this RMI metric, 40 (out of 69) clusters were modulated by either speech or limb movement tasks or both (see Table 2). A higher number of clusters were modulated by speech (*n* = 26) than limb movement (*n* = 10; Table 2). Logistic mixed effects model results indicated that this observation was statistically significant regardless of onset or offset alignment (*z* = −3.131, *p* = 0.002).

**TABLE 2:** Modulation of 69 identified neuronal clusters in response to tasks, based on RMI analyses.

	Single units	Multi units	Total
Total modulated units	17	23	40
Speech only	12	14	26
Limb only	3	7	10
Both speech and limb	2	2	4
Total non-modulated units	11	18	29
Total	28	41	69

Consistent with previous studies, the identified STN neuronal clusters demonstrated diverse patterns of modulation in neuronal firing as measured by RMIs such that some clusters showed increases in RMI, while others exhibited decreases in RMI. Table 3 displays the number of multi-unit neuronal clusters that showed significant changes in RMI in response to the onset and offset of speech and limb movement. Generally, more clusters showed increased RMIs for speech compared to limb movement.

**TABLE 3:** Modulation patterns of the 41 identified STN multi-unit clusters relative to speech and limb movement onset and offset.

	Speech onset	Speech offset	Limb onset	Limb offset
Increase in RMI	15	10	7	1
Decrease in RMI	5	3	3	3
Total	20	13	10	4

Note that since some of the 41 identified clusters (see also Table 2) were modulated by both onset and offset alignments there is a total of 47 clusters shown here.

We were also interested in the potential correlations between STN modulation and four common PD clinical variables. These variables included 3 preoperative measures of motor impairment in the OFF vs. ON levodopa state (Δ UPDRS III _total_; Δ UPDRS III _speech_; Δ UPDRS III _fingerbradykinesia_) and PD duration. First, all 69 STN clusters were examined together and showed that these clinical variables did not significantly correlate with STN firing patterns. Particularly, Δ UPDRS III _total_ did not interact with task (speech vs. limb movement) in prediction of firing rate with either RMI [*t* (204.9) = 0.26, *p* = 0.79] or absolute firing rate [*t* (66.15) = −0.18, *p* = 0.85]. Similarly, PD duration was not a predictor of STN neuronal firing rates during tasks [*t* (51.09) = 1.16, *p* = 0.25].

**FIGURE 5:**
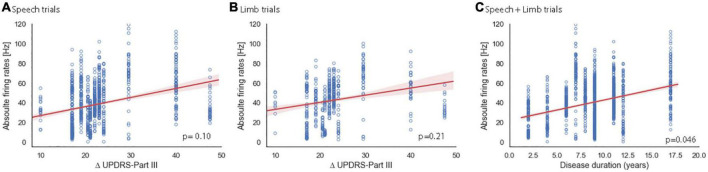
Relationship of sensitivity to dopaminergic replacement **(A,B)** and PD duration **(C)** to single unit STN firing rates. Red shaded areas illustrate the 95% confidence interval of regression analysis. Change in pre-operative total UPDRS III score in the OFF vs. ON PD medication state for speech **(A)** and limb **(B)** trials. Note that two participants had the same Δ UPDRS III _total_ and are overlapping in panels **(A,B)** at 22 (s261, s270). **(C)** PD duration showed a significant correlation with single-trial firing rate for speech and limb trials. Note that two pairs of subjects have the same PD durations and are overlapping in this plot at 6 years (s265, s270) and at 11 years (s277, s281).

A previous report documents increased STN firing rates as PD duration increases at a single-neuron level (Remple et al., 2011). Therefore, we examined our single- and multi-unit clusters separately with the 4 clinical variables. Figure 5 shows the results of correlations between firing rates and Δ UPDRS III separately for speech and limb, and PD duration for single neurons. Analyses did not show significant correlation between STN firing rates of single units and Δ UPDRS III_total_ for speech [*t* (14.42) = 1.79, *p* = 0.10; Figure 5A] or limb movement trials [*t* (14.26) = 1.31, *p* = 0.21; Figure 5B]. Consistent with the Remple study (2011), we did identify a significant positive correlation between PD duration and higher STN firing rates at a single unit level regardless of task [*t* (15.63) = 2.16, *p* = 0.046; Figure 5C]. There was no significant correlation between RMIs of speech and limb movement and clinical variables. Similarly, analysis did not show significant correlations between firing rates or RMI and clinical characteristics of PD at the multi-unit level.

## Discussion

We investigated how STN neurons were modulated by speech and limb movement, and whether firing rates during speech and limb movement can index clinical characteristics of PD such as disease duration and indices of dopaminergic sensitivity. In the present study, we identified 69 units within right and left STNs of 12 PD participants while they were performing interleaved speech and limb movements. Heterogenous neuronal activity (e.g., increasing, unchanged, and decreasing in RMIs) was observed in STN for speech and limb movement (see Table 3), consistent with previous studies (Lipski et al., 2018; London et al., 2021). Our data provides evidence that suggest STN neurons are slightly differentially modulated by speech and limb movement. First, we found that there are units within STN that are responding to only limb movement, only speech, or both tasks (see Table 2), with a higher number of neurons showing modulation by speech than limb movement. Second, findings indicated that RMIs significantly increased in response to speech vs. limb movement (see Figure 2A). Finally, we found that STN single neurons, regardless of task, had higher firing rates with increased PD duration (see Figure 3C).

We did not find any significant difference between absolute STN firing rates (i.e., Hz) during limb movement and speech in the present study. No previous studies have directly compared the firing rates of STN neurons for speech vs. limb movement. Therefore, we must compare our observed firing rates with previous studies separately reporting rates during limb movement or speech tasks.

Several studies have examined the neuronal firing patterns during limb movements. Theodosopoulos et al. (2003) found that 49% (149 out 303) of STN neurons were modulated by upper and lower limb movements with mean firing rate of 34.8 Hz for movement related neurons and 31 Hz for unresponsive units (Theodosopoulos et al., 2003). London et al. (2021) reported a diverse pattern of neuronal firing rates in response to limb movement in eight PD patients. They identified 39 single and multi-units within the STN with the mean firing rates of 36.6 Hz (London et al., 2021). The firing rates we obtained in response to limb movement was slightly higher (42.1 Hz) compared to Theodosopoulos et al.’s and London et al.’s studies. This small difference might be due to different tasks utilized across studies. We only tested neuronal firing rates during upper limb movement (i.e., finger-tapping) whereas Theodosopoulos et al. examined both upper and lower limb movement (note that they did not report the firing rates separately). Moreover, London et al. examined STN neuronal firing rates in response to a reach-to-grasp task which is a more complex motor task than our finger-tapping task. It is also worth pointing out that our task involved higher frequency limb movements, i.e., finger tapping, compared to these other studies and it is quite possible that differences in mean firing rates could be related to differences in movement frequency, akin to reports of STN firing differences with and without limb movement due to tremor (Levy et al., 2002).

Previous studies have also reported STN neuronal firing rates in response to speech tasks. Lipski et al. (2018) reported that 53% of 79 STN neurons were modulated by a visually cued consonant+vowel+consonant speech task. Watson and Montgomery (2006) reported speech-related modulation in 60% of 35 mostly right-sided STN neurons using a single sentence repetition task. Tankus and Fried (2019) have also reported STN neuronal modulation during speech. They reported that STN firing rates were significantly lower in subjects with hypophonia during overt vowel production (28 Hz) compared to subjects without hypophonia (46 Hz). However, in patients with hypophonia, the percentage of speech-related units during production was significantly higher (66%) than seen in patients without hypophonia (40%). In addition, Tankus et al. (2021) recorded a total of 180 STN units of which 69% were modulated by a speech task. They found that neural firing was delayed in patients with speech impairments relative to patients without disorders. Our subjects were not dichotomized based on presence or absence of hypophonia or any other speech impairment, but our results are consistent with those of Tankus and Fried (2019) since our reported firing rate during speech (i.e., 40.6 Hz) lies within the range of their two groups. It is also notable that our study used different cues and speech tokens (i.e., sustained vowel [/ɑ/] phonation and a diadochokinetic task [/tɑtɑtɑ/]) and analyzed different epochs (full duration vs. brief speech onset/offset periods, i.e., RMI) compared to other studies. As such, our findings apply to simple speech tokens and the generalizability of our findings to more complex speech tasks, such as connected speech (e.g., conversation, speech monolog) or complex limb tasks, merits additional study.

The inconsistency between our RMI findings (i.e., significant difference for limb vs. speech) and absolute firing rates (no significant difference for limb vs. speech) may imply that RMI is a more sensitive measure of event-related modulation compared to absolute firing rate since it is calculated based on changes relative to baseline. In addition, RMI has different temporal windows of interest compared to absolute firing rates since it primarily measures the changes in the firing rates around the task onset or offset, reflecting a briefer (i.e. phasic) type of neuronal response, whereas absolute firing rates include whole trial duration and therefore would better identify sustained or tonic type neuronal firing patterns. Both techniques are accepted measures of single and multi-unit analysis.

### Correlation between PD clinical characteristics and neuronal activity

Our observation of increased STN firing in single units of PD participants with longer disease duration is consistent with the findings of Remple et al. (2011). They report a median firing rate of 29 Hz in an “early” group (mean = 3.1 years of PD medication use) versus 36 Hz in advanced group (mean = 10.3 years of PD medication use). They report differences in OFF vs. ON UPDRS III scores in their early and advanced groups (15.1 vs. 24.8 respectively). Remple et al. only explored this correlation in single units similar to what we observed, namely a significant correlation between PD duration and absolute firing rates of single, but not multi, units. This finding implies that STN firing rates at the single unit level may be a more reliable indicator of PD severity. Future study is needed to further expand upon the relationships between STN firing rates, disease duration and sensitivity to PD medication.

### Limitation and directions for future studies:

Our study has several limitations but does provide directions for future studies. First, we have recorded from only a fraction of the STN, i.e., the dorsal portions, given that the dorsal region is accepted to represent the motor subregion and effectuates the greatest clinical improvement during chronic DBS Additional studies are also needed to interpret how our evidence of differentiated speech- and limb movement-related firing in individual neurons aligns with the current understanding of STN functional organization. It is also unclear how greatly the density of speech-modulated neurons at the STN recording site might vary across subjects. Our recording locations within STN were limited to the dorsal half of the nucleus with a combination of locations in the anterior, center, and posterior thirds of the nucleus. The number of clusters we obtained does not yet allow rigorous differentiation of these subregions. Of note, one recent study did not observe differences in speech-related STN neuronal modulation as a function of recording location (Lipski et al., 2018) while another reported improved voice function with STN-DBS application at the dorsal anterior portion of the nucleus (Jorge et al., 2020); this may be consistent with more robust speech motor representation in dorsal STN. Moreover, we and previous studies did not have a control task to confirm the speech-specificity of “speech-related” neuronal modulation we report. Speech production requires varying degrees of movement of orofacial structures, and future work should incorporate non-speech orofacial movements to differentiate between speech-specific modulation and orofacial movement-related modulation. There is evidence from previous studies demonstrating that STN neurons are modulated by non-speech facial/jaw movement (Delong et al., 1984; Abosch et al., 2002; Golshan et al., 2018). For example, Abosch et al. (2002) isolated 9 of 64 neurons within STN that were modulated by orofacial non-speech movement. Hence, we acknowledge that modulation of firing rates in STN neurons during our speech task, like all other reports to date and including both speech onset and speech offset, may not be solely induced by speech. It is reasonable to assume that orofacial movement during speech tasks may have contributed to the modulation of firing rates we report as well as previous studies reporting STN neuronal activity during speech (e.g., Lipski et al., 2018; Tankus and Fried, 2019; Tankus et al., 2021). However, we think that future studies will benefit from our findings to further elucidate the role of STN neurons in speech and limb movement.

## Data availability statement

The raw data supporting the conclusions of this article will be made available by the authors, without undue reservation.

## Ethics statement

The studies involving human participants were reviewed and approved by the University of Iowa Institutional Review Board. The patients/participants provided their written informed consent to participate in this study.

## Author contributions

JG designed the research. JG and RK performed the research. RK, JG, KJ, and JB analyzed the data. CP, RK, and KJ performed the statistical analyses. KJ, RK, and JG wrote the manuscript. All authors contributed to the article and approved the submitted version and edited the manuscript.
